# The impact of social activity on cardiovascular disease risk among middle-aged and older adults in China: a nationwide cohort study based on the CHARLS database

**DOI:** 10.3389/fpubh.2025.1554130

**Published:** 2025-04-10

**Authors:** Yingting Wu, Qi Cheng, Haiyang Song, Xinyue Gong, Sihan Wang, Kehui Xu, Lei Qin, Jing Cheng, Fei He

**Affiliations:** ^1^School of Nursing, Anhui University of Chinese Medicine, Hefei, Anhui, China; ^2^The Second Affiliated Hospital of Anhui University of Chinese Medicine, Hefei, Anhui, China; ^3^School of Integrated Chinese and Western Medicine, Anhui University of Chinese Medicine, Hefei, Anhui, China; ^4^Department of Cardiology, The Second Affiliated Hospital of Anhui Medical University, Hefei, Anhui, China

**Keywords:** social activity, cardiovascular disease, middle-aged and older adults, the China Health and Retirement Longitudinal Study, cohort study

## Abstract

**Objective:**

This study aims to examine the relationship between social activity and the risk of cardiovascular disease (CVD) in middle-aged and older adults in China.

**Methods:**

We used data from the China Health and Retirement Longitudinal Study (CHARLS) collected between 2011 and 2020. The study followed 4,099 participants aged 45 and older, all free from cardiovascular disease at baseline (2011), over a 9-year period. CVD status was self-reported by participants. Kaplan–Meier survival curves were employed to illustrate the cumulative incidence of cardiovascular events across different levels of social activity. Cox proportional hazards regression models and restricted cubic spline (RCS) were used to evaluate the association between social activity and CVD risk. Subgroup analyses were conducted to explore potential interactions between social activity and CVD risk, stratified by age, gender, education level, smoking and drinking status, number of chronic conditions, depression, and body mass index (BMI).

**Results:**

A total of 4,099 participants were included in the analysis. Over the 9-year follow-up period, 813 cardiovascular events occurred. After adjusting for age, gender, residence, education, marital status, smoking and drinking habits, chronic disease burden, depression, and BMI, each 0.1 decrease in social activity level was associated with a 7.4% increased risk of cardiovascular events (aHR, 1.074; 95% CI: 1.069–1.079).

**Conclusion:**

Social activity is significantly linked to the risk of cardiovascular disease among middle-aged and older adults in China. These findings emphasize the importance of maintaining social engagement to support cardiovascular health in this population.

## Graphical abstract

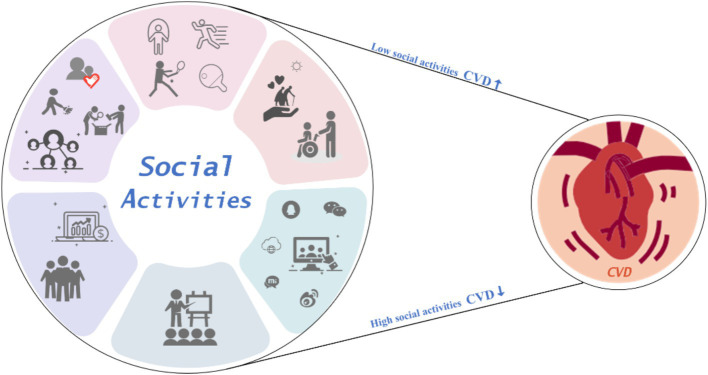

## 1 Introduction

Cardiovascular disease (CVD) continues to be a leading cause of death worldwide, representing a significant public health challenge (1, 2). In China, the prevalence of CVD is strikingly high, with an estimated 330 million people affected by the condition. This includes approximately 13 million individuals suffering from stroke and 11.39 million with coronary heart disease. The burden of CVD is expected to rise further, driven by an aging population and changing lifestyle factors (3). Although CVD incidence is also increasing among younger populations, middle-aged and older adults remain the primary target for prevention, management, and treatment due to their increased susceptibility.

Over the past few decades, considerable attention has been paid to identifying modifiable risk factors for CVD, particularly those that can be influenced through lifestyle changes. Studies conducted both in China and internationally have explored a range of factors associated with primary prevention in middle-aged and older adults, including diet, physical activity, and social participation (4–7). Among these, social activity has emerged as a key factor in promoting heart health and preventing CVD. This is because social activity is not only widely applicable but also a cost-effective way to enhance overall well-being in older adults. Social activity refers to the different ways in which individuals engage and communicate with others in their daily lives, whether through family, friends, or community interactions. Specifically, the level of social activity reflects the degree of participation in these social interactions, serving as an indicator of social connectivity and integration within society (8, 9).

Scholars generally agree that social activity plays a crucial role in social participation, contributing significantly to the physical, mental, and emotional health of older adults (10). Numerous studies have shown that active engagement in social activities among middle-aged and older adults can notably reduce the risk of chronic diseases, including cardiovascular conditions. Specifically, maintaining an active social life has been linked to lower incidence and mortality rates of cardiovascular disease, largely by improving emotional well-being and alleviating common mental health issues like anxiety and depression (11–15). In addition, social activity offers broader benefits, promoting healthier aging and easing the strain on healthcare systems by preventing a variety of age-related diseases and conditions (16). It is also associated with a better quality of life and greater longevity, making it a key focus of public health efforts aimed at meeting the needs of aging populations (17, 18).

While the growing body of literature highlights the benefits of social activity for cardiovascular health, significant gaps still exist in understanding the specific ways in which social engagement influences the risk of CVD. Many previous studies have oversimplified social activity, categorizing it as either “active” or “inactive” without considering the intensity, frequency, or different forms of social participation. This simplification may obscure the complex relationship between social engagement and cardiovascular health. Moreover, much of the existing research has relied on cross-sectional data, which only provides a snapshot of the relationship between social activity and CVD risk at one point in time (19). These types of studies are limited in their ability to establish causality, leaving open the question of whether higher levels of social engagement actually reduce the risk of cardiovascular disease, or whether individuals with lower cardiovascular risk are simply more likely to be socially active.

To address these limitations, this study draws on longitudinal cohort data from the CHARLS database, spanning from 2011 to 2020. By analyzing data from individuals aged 45 and older, the study aims to explore the association between varying levels of social activity and the long-term risk of developing cardiovascular disease. The longitudinal design allows for a more accurate examination of the causal relationship between social engagement and cardiovascular health. In doing so, the study seeks to provide stronger evidence regarding the role of social activity as a modifiable risk factor for CVD in middle-aged and older adults, offering valuable insights that can inform the development of more effective public health interventions to reduce cardiovascular disease and improve health outcomes in aging populations.

## 2 Methods

The data for this study is sourced from the CHARLS database, which is funded by Peking University. CHARLS is a comprehensive, interdisciplinary survey designed to collect high-quality, micro-level data that is broadly representative of the population aged 45 and older in China. The dataset includes a wide array of information, such as demographic details, health status, income, and asset conditions, with the primary aim of analyzing the implications of China’s aging population. Additionally, CHARLS promotes interdisciplinary research on aging-related issues and provides a scientific basis for developing and improving policies related to aging in China.

CHARLS employs a stratified sampling design coupled with probability proportional to size (PPS) sampling. The study has been conducted in several waves: 2011, 2013, 2015, 2018, and 2020. Data collection took place across 150 counties and 450 communities (or villages) in 28 provinces, autonomous regions, and municipalities. This rigorous sampling approach ensures that the CHARLS data is highly representative, capturing the conditions of middle-aged and older populations in both urban and rural areas across China. As a result, CHARLS provides a rich dataset that offers valuable insights into the demographic and health characteristics of China’s aging population.

### 2.1 Data source

**Figure 1:**
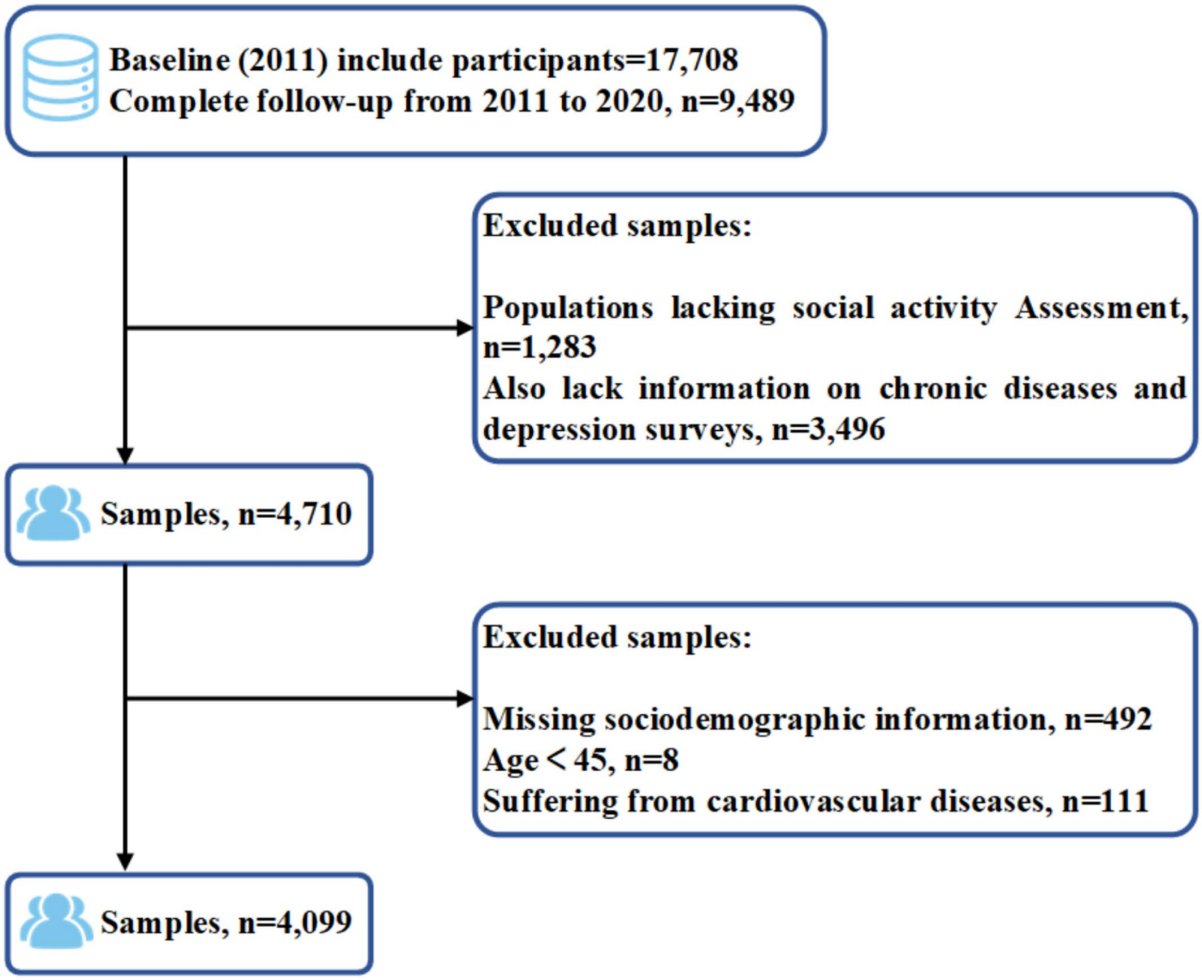
Process of participants selection.

This study uses data from five waves of the CHARLS database, specifically from the years 2011, 2013, 2015, 2018, and 2020. The baseline data from 2011 included 17,708 participants. Between 2011 and 2020, a total of 9,489 participants were followed up continuously. However, 1,283 participants were excluded due to missing data on social activity during the follow-up period, and 3,496 participants were excluded because of missing information on chronic diseases or depression assessments. Additionally, 492 participants were excluded due to missing baseline data (such as age, gender, residence, education level, and marital status), and eight participants were excluded for being younger than 45 years old. As this study follows a cohort design, participants with pre-existing cardiovascular diseases at baseline (2011) were also excluded, resulting in the removal of 111 participants. In the end, 4,099 participants remained in the analysis. The participant selection process is illustrated in Figure 1.

### 2.2 Study design

A longitudinal design was selected for this study.

### 2.3 Study variables

#### 2.3.1 Calculation of social activity

The key explanatory variable in this study is social activity. In CHARLS, information on social activity is mainly obtained through the question: “In the past month, have you participated in any of the following social activities?” This question has 12 options:

- Interacting with friends;
- Playing mahjong, chess, cards, or participating in a social club;
- Providing unpaid help to family members, friends, or neighbors who do not live with you;
- Participating in sports, social, or other types of clubs;
- Attending community-related organizations;
- Doing volunteer or charity work;
- Caring for sick or disabled adults who do not live with you and to whom you do not provide payment;
- Participating in education or training courses;
- Stock investment;
- Using the internet;
- Other activities;
- None of the above.

For each activity, the value is assigned as 1 if the participant engages in it; otherwise, it is 0.

A social activity index has been created based on the 11 social activities listed in the questionnaire and their frequency. The formula for calculating the social activity index is: $C=\overset{N=11}{\underset{i=1}{\sum }}\left(\mathrm{Ai}*Fi\right)$ . C represents the social activity index. Ai indicates whether the participant engages in a particular social activity (1 = Yes, 0 = No). Fi represents the frequency of participation in each activity (Almost daily = 3, Almost weekly = 2, Infrequent = 1) (8, 20). A higher social activity index indicates a higher level of social engagement. In this study, a score of ≤2 is classified as low social activity, and a score of≥3 is classified as high social activity.

#### 2.3.2 Cardiovascular disease assessment

The outcomes of this study are based on self-reported data used to identify new-onset cardiovascular diseases during the follow-up period. CVD is assessed by asking participants two questions: “Have you ever been diagnosed by a doctor with heart disease, coronary artery disease, angina, congestive heart failure, or other heart problems?” and/or “Have you ever been diagnosed by a doctor with a stroke?” Participants who answer “yes” to any of these conditions are assigned a score of “1,” while those who answer “no” are assigned a score of “0.” New-onset cardiovascular disease is the primary outcome in this study, with heart disease and stroke serving as secondary outcomes.

#### 2.3.3 Assessment of covariates

The selection of covariates was based on the clinical context and previous research (21). In this study, the covariates include age (<60 years, ≥60 years), gender, smoking status, alcohol consumption, education level (primary school or below, middle school, and high school or above), residential area (urban or rural), marital status (married, unmarried [including separated, divorced, widowed, and never married]), body mass index (BMI), depression, and other chronic health conditions. The chronic conditions considered include 13 categories: hypertension; dyslipidemia (elevated low-density lipoprotein, triglycerides, and total cholesterol, or reduced high-density lipoprotein levels); diabetes or hyperglycemia; cancer or malignant tumors (excluding minor skin cancers); chronic lung diseases (such as chronic bronchitis and emphysema, excluding tumors or cancer); liver diseases (excluding fatty liver, tumors, and cancer); stroke; kidney diseases (excluding tumors and cancer); gastrointestinal or other digestive diseases (excluding tumors or cancer); emotional, neurological, or psychiatric disorders; memory-related conditions (such as dementia, brain atrophy, and Parkinson’s disease); arthritis or rheumatic diseases; and asthma. Participants were asked if they had been diagnosed with any of these conditions by a doctor, and those who confirmed the diagnosis were assigned a score of 1, while those who did not report the condition received a score of 0. The total number of chronic conditions for each participant was then calculated by summing these scores. Appendix 1 shows the code for all variables and their problem descriptions.

### 2.4 Statistics

The database merging, sample selection, and data analysis were conducted using STATA 17 and SPSS 26. Continuous variables were expressed as median (interquartile range) or mean (x̅ ± SD), while categorical variables were presented as frequencies and percentages. To explore the differences between groups with varying levels of social activity, chi-square tests (χ^2^) were used to analyze the categorical variables across the groups and compare baseline characteristics of individuals in each group. A *p*-value of <0.05 was considered statistically significant.

Univariate and multivariate Cox regression analyses were employed to evaluate both crude and adjusted hazard ratios (aHRs) for social activities and other key covariates. This assessment aimed to determine the relationship between each covariate and the risk of CVD. The follow-up time for each participant was calculated from the baseline survey date (June 1, 2011) to the first occurrence of a CVD event, death, loss to follow-up, or the end of the follow-up period (June 1, 2020), whichever occurred first. The incidence rate of CVD events per 1,000 person-years was calculated. A Cox proportional hazards regression model, with hazard ratios (HRs) and 95% confidence intervals (CIs), was used to examine the association between social activity and CVD incidence. The covariates included: age and sex (Model 1); Model 1 plus residential area, education, marital status, smoking status, and alcohol consumption (Model 2); Model 2 plus comorbid chronic diseases, depression, and BMI (Model 3). restricted cubic spline (RCS) analysis was utilized to investigate potential non-linear relationships between these variables. Additionally, stratified analyses were conducted to explore whether the association between social activity and the risk of CVD incidence differed across subgroups based on sex, age, education level, marital status, residential area, smoking, alcohol use, number of comorbid chronic diseases, depression, and BMI, in order to assess potential interactions. Taking into account the potential effects of unmeasured confounders, we calculated the minimum E-value necessary to quantify the impact of these confounders on the outcome based on the final model.

## 3 Results

### 3.1 Baseline characteristics of older adults by social activity levels

This study included a total of 4,099 older adults, consisting of 1,896 males (46.3%) and 2,200 females (53.7%). The average age of all participants was 57.28 ± 7.65 years. Table 1 summarizes the baseline characteristics of participants, grouped by their social activity levels. At baseline, 1,867 participants (45.5%) were classified in the low social activity group, while 2,232 participants (54.5%) were placed in the high social activity group. To examine the relationship between individual characteristics and social activity levels, χ^2^ tests were performed to compare differences between the two groups. The results showed significant differences (all *p* < 0.05) between the low and high social activity groups in terms of age, residential area, marital status, education level, alcohol consumption, depression, and BMI.

**Table 1:** Baseline characteristics of participants by social activity levels.

Variables	Total (*n* = 4,099)	Social activity subgroups		*p* value
		Low (*n* = 1867)	High (*n* = 2,232)	
Age				
<60	2,662 (64.9)	1,155 (43.4)	1,507 (56.6)	<0.001
≥ 60	1,437 (35.1)	712 (49.6)	725 (50.4)	
Gender				
Male	1896 (46.3)	858 (45.3)	1,038 (54.7)	0.725
Female	2,203 (53.7)	1,009 (45.8)	1,194 (54.2)	
Resident				
Urban	2,277 (55.6)	724 (31.8)	1,553 (68.2)	<0.001
Rural	1822 (44.4)	1,143 (62.7)	679 (37.3)	
Marital status				
Married	3,741 (91.3)	1,682 (45.0)	2059 (55.0)	0.015
Unmarried	358 (8.7)	185 (51.7)	173 (48.3)	
Education level				
Primary school and below	2,728 (66.6)	1,327 (48.6)	1,401 (51.4)	<0.001
Middle school	890 (21.7)	370 (41.6)	520 (58.4)	
High school and above	481 (11.7)	170 (35.3)	311 (64.7)	
Smoking				
Yes	1,560 (38.1)	705 (45.2)	855 (54.8)	0.720
No	2,539 (61.9)	1,162 (45.8)	1,377 (54.2)	
Drinking				
Yes	1,394 (34.0)	600 (43.0)	794 (57.0)	0.021
No	2,705 (66.0)	1,267 (46.8)	1,438 (53.2)	
Number of chronic diseases				
0	433 (10.6)	208 (48.0)	225 (52.0)	0.059
1	2031 (49.5)	953 (46.9)	1,078 (53.1)	
2	1,061 (25.9)	448 (42.2)	613 (57.8)	
≥ 3	574 (14.0)	258 (44.9)	316 (55.1)	
Depression symptoms				
No	1,679 (40.9)	796 (47.4)	883 (52.6)	0.046
Yes	2,420 (59.1)	1,071 (44.3)	1,349 (55.7)	
BMI				
Normal Weight	2061 (50.3)	905 (43.9)	1,156 (56.1)	0.019
Underweight	198 (4.8)	103 (52.0)	95 (48.0)	
Overweight	1,287 (31.4)	619 (48.1)	668 (51.9)	
Obese	553 (13.5)	240 (43.4)	313 (56.6)	

BMI, body mass index.

### 3.2 Univariate and multivariate cox proportional hazards regression analysis of factors affecting CVD risk

Table 2 presents the results of the univariate analysis, indicating that the risk of CVD was not significantly associated with residence, education level, or smoking (*p* > 0.05). However, it exhibited a positive correlation with age, gender, marital status, the number of chronic diseases, depression, and BMI, while showing a negative correlation with alcohol consumption and social activity (all *p* < 0.005). To further investigate the relationships between CVD risk and these factors, we adjusted for confounding variables. The adjusted results demonstrated that CVD risk remained positively associated with age, gender, the number of chronic diseases, depression, and BMI, and negatively associated with alcohol consumption and social activity (all *p* < 0.05). In contrast, marital status did not achieve statistical significance after adjustment (*p* = 0.213) (Supplementary Table S1). Consequently, female, aged 60 or older, the presence of one or more chronic diseases, depression symptoms, and overweight or obesity were identified as high-risk factors for CVD, whereas alcohol consumption and high levels of social activity were identified as protective factors against CVD.

**Table 2:** Univariate Cox proportional hazards regression analysis of factors affecting the risk of CVD.

Variables	All participants	(HR, 95%CI)	*p* value
Age			
<60	2,662 (64.9)	Ref	
≥ 60	1,437 (35.1)	1.289 (1.121, 1.483)	<0.001
Gender			
Male	1896 (46.3)	Ref	
Female	2,203 (53.7)	1.233 (1.073, 1.418)	0.003
Resident			
Urban	2,277 (55.6)	Ref	
Rural	1822 (44.4)	1.107 (0.961, 1.275)	0.160
Marital status			
Married	3,741 (91.3)	Ref	
Unmarried	358 (8.7)	1.309 (1.104, 1.552)	0.002
Education level			
Primary school and below	2,728 (66.6)	Ref	
Middle school	890 (21.7)	0.929 (0.781, 1.105)	0.406
High school and above	481 (11.7)	1.033 (0.835, 1.279)	0.763
Smoking			
No	2,539 (61.9)	Ref	
Yes	1,560 (38.1)	0.915 (0.793, 1.056)	0.223
Drinking			
No	2,705 (66.0)	Ref	
Yes	1,394 (34.0)	1.422 (1.220, 1.657)	<0.001
Number of chronic diseases			
0	433 (10.6)	Ref	
1	2031 (49.5)	1.806 (1.537, 2.122)	<0.001
2	1,061 (25.9)	3.117 (2.540, 3.826)	<0.001
≥ 3	574 (14.0)	4.742 (3.680, 6.112)	<0.001
Depression symptoms			
No	1,679 (40.9)	Ref	
Yes	2,420 (59.1)	1.585 (1.381, 1.819)	<0.001
BMI			
Normal Weight	2061 (50.3)	Ref	
Underweight	198 (4.8)	1.061 (0.730, 1.542)	0.755
Overweight	1,287 (31.4)	1.285 (1.099, 1.502)	0.002
Obese	553 (13.5)	1.650 (1.362, 1.999)	<0.001
Social activity			
Low	1960 (47.8)	Ref	
High	2,139 (52.2)	0.331 (0.285, 0.386)	<0.001

HR, hazards ratio; CI, confidence interval, BMI, body mass index.

### 3.3 Association between cardiovascular disease incidence and social activity level

After a follow-up period of 9 years, 813 out of 4,099 participants (19.8%) developed CVD, including 609 cases of heart disease (14.9%) and 283 cases of stroke (6.9%). Among participants with low social activity, 581 cases (71.5%) experienced cardiovascular events, whereas 232 cases (28.5%) were reported in the high social activity group. The incidence rate of CVD was 35.1 per 1,000 person-years in the low social activity group, compared to 12.32 per 1,000 person-years in the high social activity group. This demonstrates a significantly higher risk of CVD in participants with low social activity, a trend also observed for both heart disease and stroke.

To further examine the relationship between social activity level and CVD risk, three Cox proportional hazards regression models were applied (Table 3). After adjusting for potential confounders in Model 3, participants with high social activity were found to have an 85.1% lower risk of developing CVD compared to those with low social activity (adjusted hazard ratio [aHR] 0.149; 95% confidence interval [CI], 0.120–0.183). Additionally, when social activity was treated as a continuous variable, every 0.1 decrease in social activity was associated with a 7.4% increase in CVD risk (aHR 1.074; 95% CI, 1.069–1.079).

**Table 3:** Association between social activity level and CVD risk.

Outcome	Cases, No.	Incidence rate, per 1,000 person-years	HR (95%CI)			*p* value
			Model 1	Model 2	Model 3	
CVD (*n* = 813)						
ACT group						
Low	581	35.10	1	1	1	
High	232	12.32	0.340 (0.291, 0.397)	0.117 (0.095, 0.145)	0.149 (0.120, 0.183)	*p* < 0.001
Per 0.1 decrement			1.080 (1.075, 1.085)	1.072 (1.067, 1.077)	1.074 (1.069, 1.079)	
Heart disease (*n* = 609)						
ACT group						
Low	435	26.29	1	1	1	
High	174	9.24	0.346 (0.290, 0.414)	0.108 (0.085, 0.137)	0.131 (0.103, 0.167)	*p* < 0.001
Per 0.1 decrement			1.080 (1.075, 1.085)	1.069 (1.064, 1.074)	1.071 (1.066, 1.076)	
Stroke (*n* = 283)						
ACT group						
Low	204	12.33	1	1	1	
High	79	4.19	0.329 (0.253, 0.428)	0.134 (0.093, 0.193)	0.195 (0.137, 0.275)	*p* < 0.001
Per 0.1 decrement			1.084 (1.079, 1.089)	1.081 (1.076, 1.086)	1.082 (1.077, 1.087)	

HR, hazards ratio; CI, confidence interval; CVD, cardiovascular disease. Model 1: adjusted for age and gender. Model 2: adjusted for age, gender, resident, marital status, educational level, smoking and drinking. Model 3: adjusted for age, gender, resident, marital status, educational level, smoking, drinking, Number of chronic diseases, Depression symptoms and BMI (body mass index).

**Figure 2:**
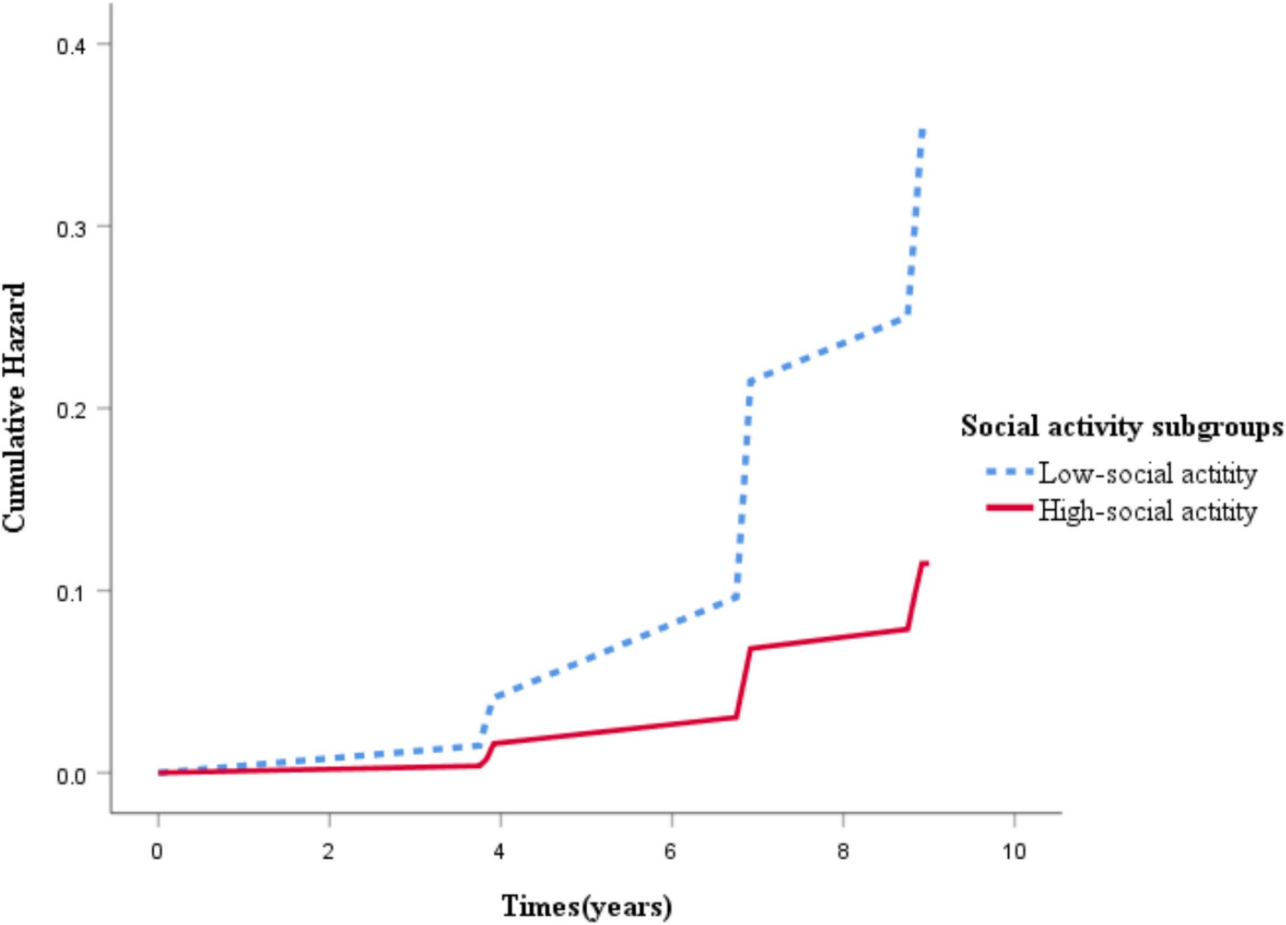
Kaplan–Meier curves of cumulative cardiovascular disease incidence in groups with different social activity levels.

Figure 2 displays the Kaplan–Meier curves for the cumulative incidence of CVD among all study participants, while additional files (Supplementary Figures S1, S2) present the Kaplan–Meier curves for heart disease and stroke. The results indicate that the cumulative incidence of CVD in the low social activity group increased progressively, and was significantly higher compared to the high social activity group.

### 3.4 Nonlinear relationship between social activity level and cardiovascular disease risk

**Figure 3:**
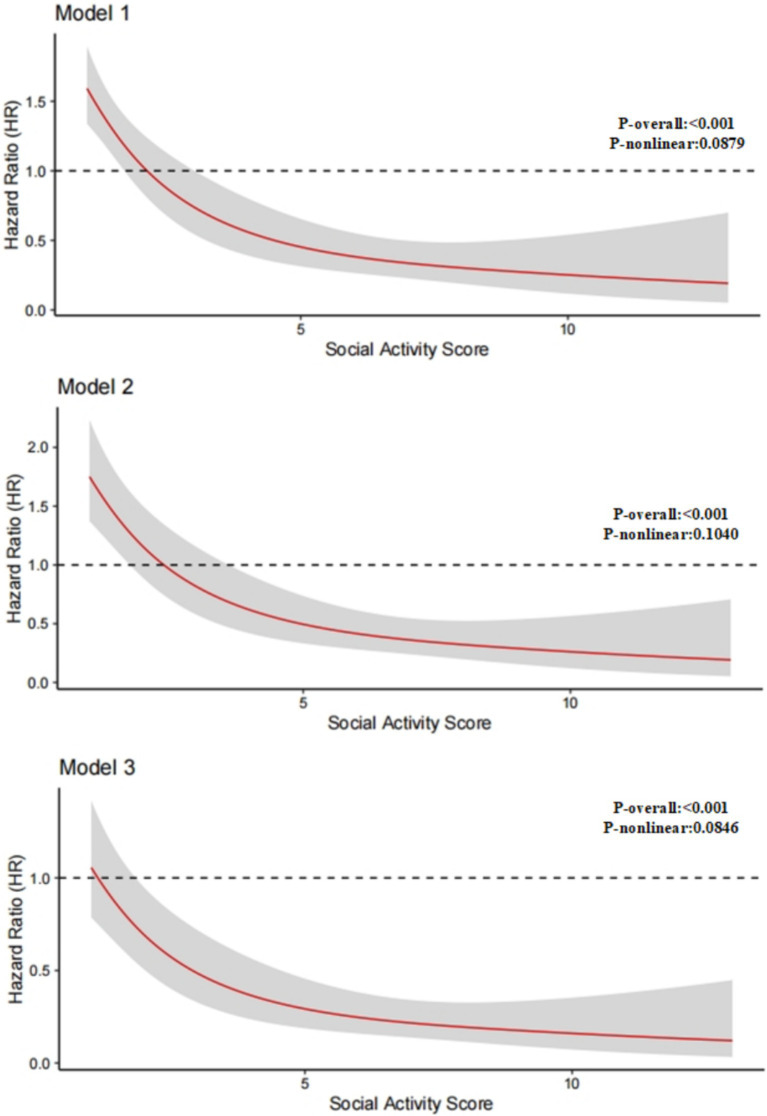
Nonlinear relationship between social activity level and cardiovascular disease risk.

To further investigate the relationship between social activity level and the risk of CVD, we employed restricted cubic spline (RCS) modeling to examine the potential non-linear relationship between total social activity scores and CVD across three different models. These models take into account various confounding factors. The results consistently demonstrated a significant negative linear relationship between social activity and the risk of CVD incidence (Figure 3). Specifically, the association between social activity and CVD risk remained robust across all models (*p*-overall <0.001), with no evidence of a significant non-linear pattern (*p*-nonlinear >0.05 in all models, ranging from 0.0846 to 0.1046). These findings suggest that as participation in social activities increases, the risk of CVD incidence progressively decreases.

### 3.5 Sub-group analysis

**Figure 4:**
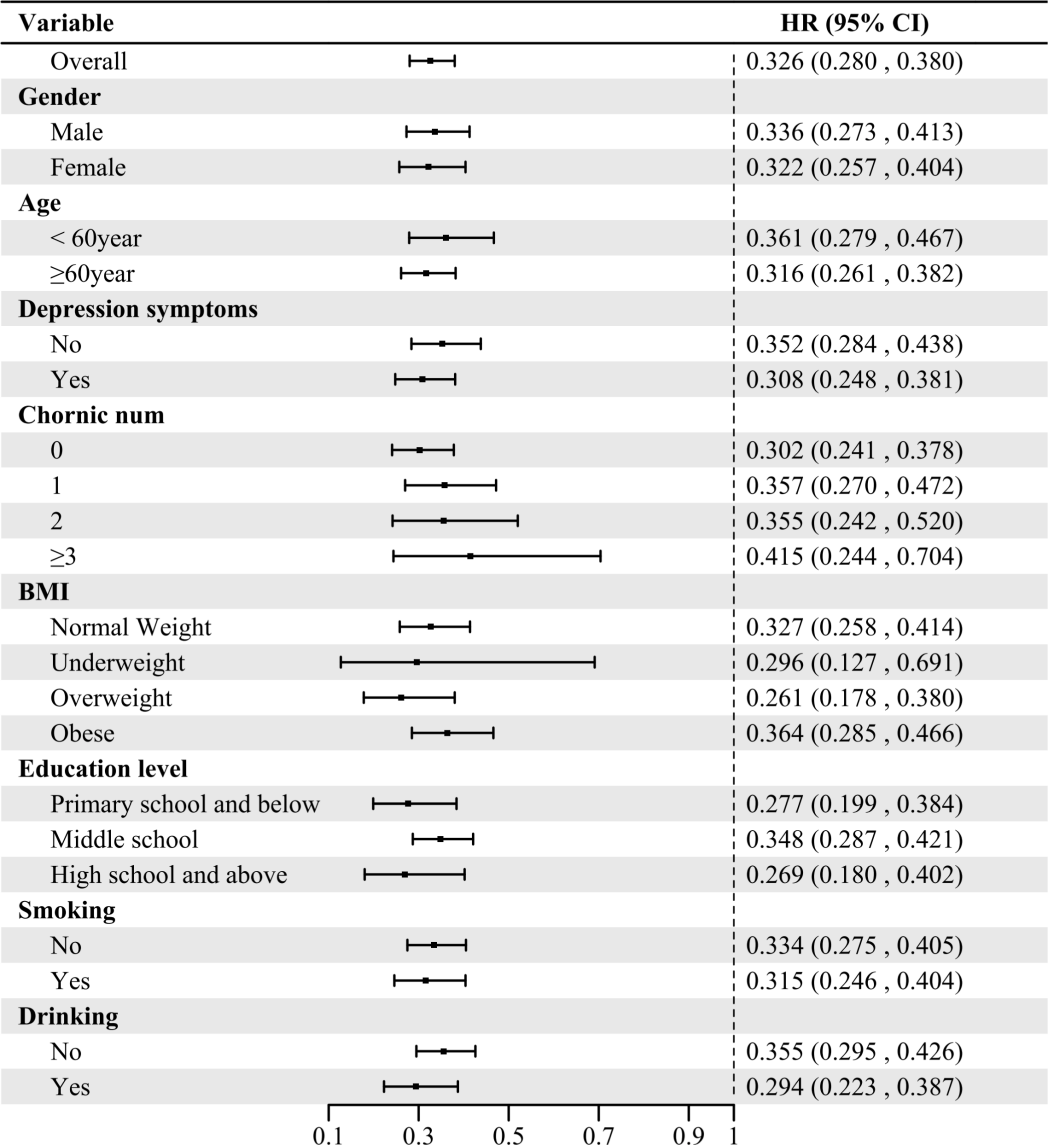
Association between social activity level and cardiovascular disease incidence stratified by different factors. HR, hazards ratio; CI, confidence interval; BMI, body mass index.

To further investigate the relationship between social activity level and CVD risk, we performed a series of subgroup analyses. As shown in Figure 4, the associations between social activity level and CVD risk were not significantly influenced by factors such as age, gender, education level, smoking status, alcohol consumption, the number of comorbid chronic diseases, depression, or BMI. Subsequently, adjustments for age, gender, education level, smoking status, alcohol consumption, number of chronic disease, depression, and BMI were made. Supplementary Table S2 shows the analysis of social activity level and CVD risk stratified by age, gender, education level, smoking status, alcohol consumption, number of chronic disease, depression, and BMI. No significant interaction was observed between these variables and social activity level (interaction *p* > 0.05), indicating a consistent association across subgroups. Finally, To evaluate the potential impact of unmeasured confounding on the results, we calculated the E-value. These substantial E-values suggest that an unmeasured confounder would need to exhibit an association strength of 12.902 (for the HR) or 16.150 (for the CI) with both the exposure and the outcome to render the observed association non-significant. Consequently, the study results are robust against potential unmeasured confounding.

## 4 Discussion

CVD is a major global health challenge, placing significant strain on healthcare systems worldwide. In the context of advancing the “Healthy China” strategy, it is critical to reduce the disease burden associated with CVDs. The World Health Organization (WHO) has emphasized the importance of modifiable social determinants of health in mitigating the risk of CVD (17). Social activity, as a simple, universally applicable, and cost-effective strategy, has been shown to reduce both the incidence and mortality of CVDs, particularly among middle-aged and older adults populations (22). Consequently, promoting active participation in social activities among these age groups is essential for fostering healthy aging and optimizing public health resource allocation (18, 23).

This study used cohort data from the China Health and Retirement Longitudinal Study (CHARLS) covering the period from 2011 to 2020. The sample consisted of 4,099 participants aged 45 and older, and the follow-up period was nine years, during which 813 participants developed cardiovascular disease. After adjusting for potential confounders, a decrease of 0.1 in social activity was associated with a 7.4% increase in CVD risk (aHR 1.074; 95% CI, 1.069–1.079). Both the restricted cubic spline analysis and the Kaplan–Meier survival curves revealed a strong association between social activity levels and the incidence of cardiovascular disease. Specifically, social activity was negatively correlated with the risk of CVD, indicating that lower levels of social activity were associated with a higher risk of cardiovascular disease, while higher levels of social engagement were linked to lower risks. These findings align with those of Liu et al. (24) As a result, social activity appears to be a significant protective factor against CVD, and healthcare professionals should evaluate the social activity levels of middle-aged and older adults individuals to enhance prevention strategies.

At baseline, 45.5% of participants (1,867 individuals) reported low levels of social activity, suggesting that social engagement is generally low among the middle-aged and older adults population in China. This finding is consistent with previous research by Yang et al. (8) The social activities considered in this study primarily included physical activity and the exchange of social information.

From the perspective of physical activity, regular engagement in physical activity can enhance cardiorespiratory fitness and improve vascular elasticity, thereby reducing the risk of CVD. Major risk factors for cardiovascular diseases, such as hypertension, dyslipidemia, and overweight/obesity, can be mitigated through physical activity (19, 25). Studies by Du et al. have shown that moderate to high levels of physical activity significantly reduce the risk of developing CVDs (26). Additionally, Fu et al. found that a lack of physical activity is closely associated with the incidence of obesity, hypertension, and other cardiovascular conditions (27). Research also suggests that even light physical activities, such as household chores or cycling, can reduce the risk of coronary heart disease (28). However, contrary to the findings of Dong et al., which suggest that physical activity may extend the lifespan of individuals with existing cardiovascular conditions, its role in preventing these diseases appears to be limited (29).

From the perspective of social information exchange, participation in social activities provides access to external health resources for middle-aged and older adults individuals. Those who engage in social activities more frequently tend to acquire more health-related knowledge and become more aware of their own health status. As a result, they are more likely to adopt proactive health behaviors, such as quitting smoking or reducing sedentary behavior, thereby lowering their risk of CVDs (30). American research has similarly found that social engagement, including volunteering, significantly reduces the incidence of CVDs (31). In addition, in Western countries, religious activities are often viewed as a form of group engagement. By offering social support and alleviating negative emotions, these activities can have a positive impact on cardiovascular health (32, 33). This study also identified depression as an independent risk factor for cardiovascular diseases in middle-aged and older adults individuals. Patients often neglect their health due to negative emotional states, leading to a diminished awareness of disease risk, consistent with the findings of Zhang et al. (34). Furthermore, research conducted by Greek scholars (35, 36) has demonstrated that individuals with depression are more likely to engage in unhealthy behaviors and face an increased risk of cardiovascular disease. However, spirituality—encompassing religiousness and spiritual experiences—can act as a protective factor for mental health by alleviating stress and fostering positive emotions. Therefore, encouraging middle-aged and older adults individuals to participate in a variety of social and recreational activities can improve their mental health and potentially reduce the risk of cardiovascular diseases. Longitudinal studies by Bergum et al. support this, showing that active social participation positively impacts mental health and can reduce the risk of CVDs (37).

Several risk factors for CVD were identified in this study, including female gender, advanced age, the presence of chronic diseases, and being overweight or obese. These findings differ somewhat from those reported by Zhang et al., who found that males, older individuals, and those who are overweight or obese have a higher risk of cardiovascular diseases (38). In our study, female gender emerged as a significant risk factor, which may be due to the predominance of postmenopausal women in the sample (39). Therefore, healthcare professionals should carefully assess the health behaviors of women and encourage their participation in social activities to reduce the risk of CVD.

Furthermore, the study revealed that moderate alcohol consumption might have a protective effect against cardiovascular diseases, aligning with the findings of Shi et al. (40). This protective effect may be attributed to the cardioprotective properties of estrogen. Both domestic and international studies have suggested that moderate alcohol consumption may reduce the risk of cardiovascular damage (41). However, it is important to consider individual health conditions and regional drinking practices when assessing this factor. A personalized approach, taking into account age, gender, and overall health, is necessary for making appropriate recommendations regarding alcohol consumption. Furthermore, the subgroup analysis conducted in this study bolsters the credibility of our evaluation regarding the relationship between social activity and the risk of cardiovascular diseases.

This study presents several limitations. First, both CVD outcomes and social activity levels were based on self-reported data, which may introduce recall bias. Second, although we adjusted for multiple confounding factors, there may still be unmeasured confounding variables (such as religious beliefs, biological markers, and others) that could impact the accuracy of the results. Furthermore, the adjusted hazard ratio (HR) being lower than the crude HR suggests the potential influence of negative confounding factors. Future studies are necessary to further validate the association between social activity and the risk of cardiovascular disease. Lastly, this study focused primarily on the middle-aged and older adults population in China (aged 45 and above), and further research is needed to determine whether these findings are applicable to other age groups or populations in different countries.

## 5 Conclusion

The findings of this study demonstrate a significant correlation between social activity and the risk of cardiovascular diseases in the middle-aged and older adults population in China. Increasing social activity among these demographics may play a crucial role in the primary prevention of cardiovascular diseases.

## Data Availability

The original contributions presented in the study are included in the article/Supplementary material, further inquiries can be directed to the corresponding authors.
