# Supplementary material for: The impact of social activity on cardiovascular disease risk among middle-aged and older adults in China: a nationwide cohort study based on the CHARLS database
Source: Front Public Health. 2025 Apr 10;13:1554130. doi: 10.3389/fpubh.2025.1554130 (PMC12018239; doi:10.3389/fpubh.2025.1554130)
Supplement: Supplementary file 1 [file Data_Sheet_1.PDF]

Supplementary Material

The Impact of Social Activity on Cardiovascular Disease Risk Among Middle-Aged and Older Adults in China: A Nationwide Cohort Study Based on the CHARLS Database

1 Supplementary Figures

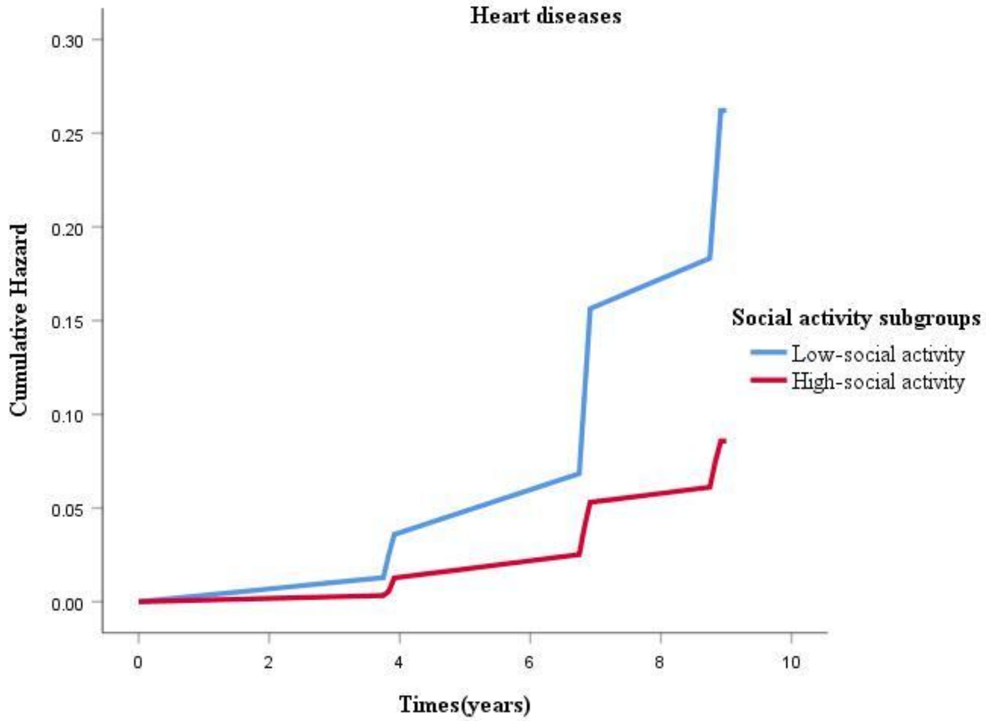

SFigure 1 Kaplan-Meier Curves of Cumulative Heart Disease Incidence in Groups with Different Social Activity Levels

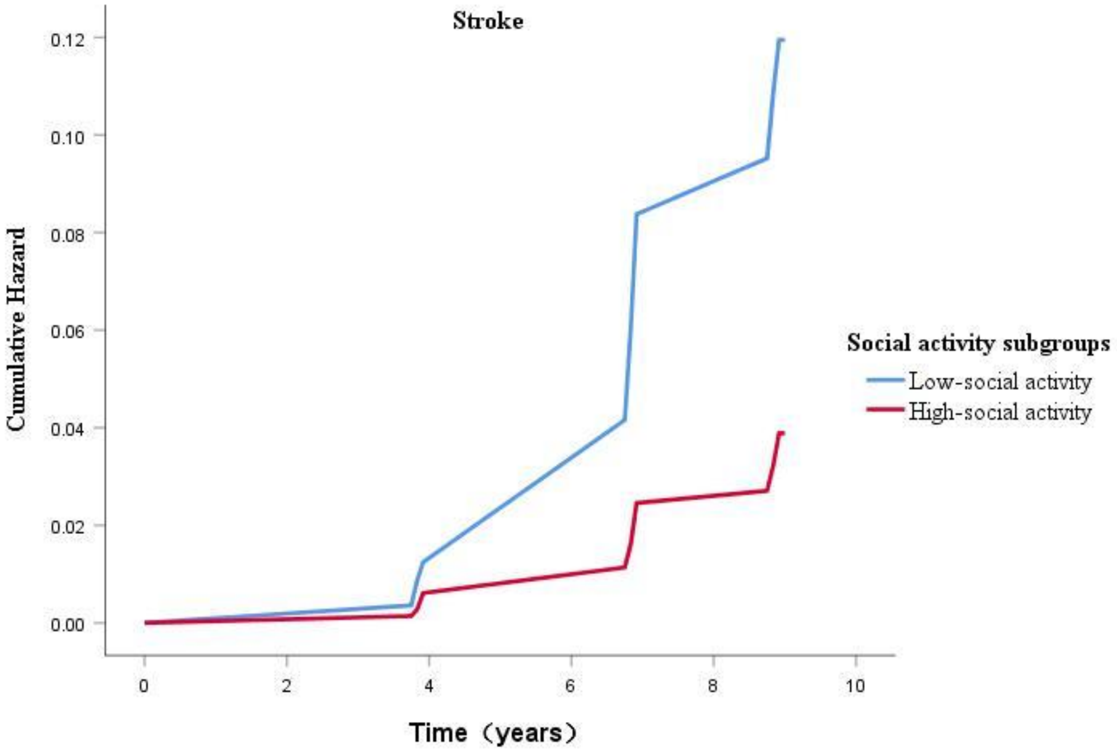

SFigure 2 Kaplan-Meier Curves of Cumulative Stroke Incidence in Groups with Different Social Activity Levels

2 Supplementary Tables

Table S1 Multivariate Cox Proportional Hazards Regression Analysis of Factors Affecting the Risk of CVD

Variables	All participants	(HR, 95%CI)	*P* value
Social activity			
Low	1960 (47.8)	Ref	
High	2139 (52.2)	0.148(0.120, 0.183)	＜0.001
Age			
＜60	2662 (64.9)	Ref	

≥60	1437（35.1）	1.174(1.017, 1.356)	0.029
Gender			
Male	1896（46.3）	Ref	
Female	2203（53.7）	1.187(1.035, 1.362)	0.014
Marital status			
Married	3741（91.3）	Ref	
Unmarried	358（8.7）	1.123(0.936, 1.347)	0.213
Drinking			
No	2705（66.0）	Ref	
Yes	1394（34.0）	0.809(0.683, 0.959)	0.014
Number of chronic disease			
0	433 (10.6)	Ref	
1	2031 (49.5)	1.598(1.359, 1.880)	＜0.001
2	1061 (25.9)	2.538(2.058, 3.129)	＜0.001
≥3	574 (14.0)	3.018(2.394, 4.035)	＜0.001
Depression symptoms			
No	1679 (40.9)	Ref	

Yes	2420 (59.1)	1.324(1.147, 1.530)	＜0.001
BMI			
Normal Weight	2061 (50.3)	Ref	
Underweight	198 (4.8)	0.918(0.630, 1.337)	0.655
Overweight	1287 (31.4)	1.276(1.088, 1.497)	0.003
Obese	553 (13.5)	1.567(1.287, 1.907)	＜0.001

Abbreviation: HR, hazards ratio; CI, confidence interval, BMI, body mass index. The above model was adjusted for social activity, age, gender, marital status, alcohol consumption, depression, number of chronic diseases, and BMI.

Table S2 Stratified associations between social activity level and CVD risk

Variables	Total（n=4099）	Social activity subgroups, HR (95% CI) *P* value		*P* for interaction
		Low（n=1867）	High（n=2232）	
Age				
＜60	2662	Ref	0.140 (0.108, 0.182) p<0.001	0.431
≥60	1437	Ref	0.158 (0.111, 0.226) p<0.001	
Gender				

Male	1896	Ref	0.129 (0.095, 0.177) p<0.001	0.791
Female	2203	Ref	0.162 (0.122, 0.215) p<0.001	
Education level				
Primary school and below	2728	Ref	0.148 (0.114, 0.192) p<0.001	0.357
Middle school	890	Ref	0.143 (0.091, 0.223) p<0.001	
High school and above	481	Ref	0.165 (0.097, 0.283) p<0.001	
Smoking				
Yes	1560	Ref	0.128 (0.091, 0.179) p<0.001	0.718
No	2539	Ref	0.159 (0.122, 0.207) p<0.001	
Drinking				
Yes	1394	Ref	0.121 (0.083, 0.176) p<0.001	0.278
No	2705	Ref	0.163 (0.126, 0.210) p<0.001	
Number of chronic disease				
0	433	Ref	0.095 (0.068, 0.131) p<0.001	0.379
1	2031	Ref	0.134 (0.091, 0.198) p<0.001	

2	1061	Ref	0.226 (0.141, 0.363) p<0.001	
≥3	574	Ref	0.398 (0.201, 0.787) 0.008	
Depression symptoms				
No	1679	Ref	0.126 (0.092, 0.171) p<0.001	0.418
Yes	2420	Ref	0.171 (0.128, 0.227) p<0.001	
BMI				
Normal Weight	2061	Ref	0.126 (0.092, 0.174) p<0.001	0.544
Underweight	198	Ref	0.186 (0.054, 0.635) 0.007	
Overweight	1287	Ref	0.173 (0.122, 0.244) p<0.001	
Obese	553	Ref	0.171 (0.100, 0.292) p<0.001	

Abbreviation: HR,hazards ratio; CI, confidence interval; BMI, body mass index. The above model was adjusted for age, gender, Education level, smoking status, drinking status, Number of chronic disease, Depression symptoms and BMI. In every instance, the model was not adjusted for the stratification variable.

Appendix 1 The code for all variables and their problem descriptions

Variable	Code	Question description	Answer
Gender	BA000_W2_3	Interviewer record the Respondent's gender	1. Male<br>2. Female

Age	BA002	What’s your actual date of birth?	
Resident	BB006	Where did You Mainly Live before 16	1. City / Town<br>2. Village
Highest level of education	BD001	What’s the highest level of education you have attained?	1. No formal education (illiterate)<br>2. Did not finish primary school<br>3. Sishu/home school<br>4. Elementary school<br>5. Middle school<br>6. High school<br>7. Vocational school<br>8. Two-/Three-Year College/Associate degree<br>9. Four-Year College/Bachelor’s degree<br>10. Master’s degree<br>11. Doctoral degree/Ph.D.
Marital status	BE001	What is your marital status?	1. Married and live with spouse<br>2. Married but don’t living with spouse temporarily for reasons such as work<br>3. Separated, don’t live together as a couple anymore<br>4. Divorced<br>5. Widowed<br>6. Never married

Smoking status	DA059	Have you ever chewed tobacco, smoked a pipe, smoked self-rolled cigarettes, or smoked cigarettes/cigars?	1. Yes<br>2. No
	DA061	Do you still have the habit or have you totally quit?	1. Still have<br>2. Quit
Drinking status	DA067	Did you drink any alcoholic beverages, such as beer, wine, or liquor in the past year? How often?	1. Drink more than once a month<br>2. Drink but less than once a month<br>3. None of these
Social activity participation	DA056	Have you done any of these activities in the last month? (Check all that apply)	1. Interacted with friends<br>2. Played Ma-jong, played chess, played cards, or went to community club<br>3. Provided help to family, friends, or neighbors who do not live with you<br>4. Went to a sport, social, or other kind of club<br>5. Took part in a community-related organization<br>6. Done voluntary or charity work<br>7. Cared for a sick or disabled adult who does not live with you<br>8. Attended an educational or training course<br>9. Stock investment

			10. Used the Internet<br>11. Other<br>12. None of these
Height	PI001_QI002	Can you understand the measurement method and are you willing to participate in this measurement?	1. YES<br>2. No
Weight	PL002_QL002	Can you understand the measurement method and are you willing to participate in this measurement?	1. Yes<br>2. No
Chronic disease	DA007	Have you been diagnosed with [conditions listed below, read one by one] by a doctor?	1. Hypertension<br>2. Dyslipidemia<br>3. Diabetes or high blood sugar<br>4. Cancer or malignant tumor<br>5. Chronic lung diseases<br>6. Liver disease<br>7. Heart attack<br>8. Stroke<br>9. Kidney disease<br>10. Stomach or other digestive diseases<br>11. Emotional, nervous, or psychiatric problems<br>12.Memory-related disease<br>13. Arthritis or rheumatism<br>14. Asthma
Depression	DC009	I was bothered by things that don't usually bother me	1. Rarely or none of the time

DC010	I had trouble keeping my mind on what I was doing	2. Some or a little of the time
DC011	I felt depressed	3. Occasionally or a moderate amount of the time
DC012	I felt everything I did was an effort	4. Most or all of the time
DC013	I felt hopeful about the future	
DC014	I felt fearful	
DC015	My sleep was restless	
DC016	I was happy	
DC017	I felt lonely	
DC018	I could not get “going”	
